# A comparative transcriptomics analysis of mammalian and non-mammalian acute kidney injury (AKI) models

**DOI:** 10.3389/fcell.2025.1653967

**Published:** 2025-09-26

**Authors:** Matthew R. Hawkins, Diana Cervera, Tiffany M. Tang, Rebecca A. Wingert

**Affiliations:** University of Notre Dame, South Bend, IN, United States

**Keywords:** acute kidney injury, comparative transcriptomics, regeneration, zebrafish, spiny mouse, axolotl, nephron

## Abstract

**Introduction:**

Acute kidney injury (AKI) is a complex clinical condition characterized by decline in renal function and widespread transcriptional dysregulation. While transcriptomic studies that compare human and other mammalian models of AKI have provided insight, much less is known about commonalities and contrasts between mammalian models and nontraditional and regenerative laboratory models. Understanding these molecular level responses to injury in both regenerative and non-regenerative models may reveal conserved and unique pathways in renal repair.

**Methods:**

To investigate transcriptional responses to AKI across species, we incorporated newly available RNA-seq data from zebrafish, axolotl, and spiny mouse models into an expanded cross-species comparative analysis. These were analyzed alongside existing and previously analyzed data from both human and mouse (Mus) models. Differential gene expression and gene-ontology (GO) enrichment analyses we utilized to identify conserved and regeneration-specific during injury and recovery phases.

**Results:**

Comparative transcriptomic analysis revealed distinct transcriptional programs in each species during AKI, including both shared and species-specific responses. Of note, zebrafish show differential expression of apolipoproteins, molecules of increasing interest to the greater field of nephrology. In the recovery setting, we show that animals with regenerative capacity have conserved and divergent transcriptional programs.

**Discussion:**

Our findings demonstrate that non-traditional animal models of AKI, such as zebrafish, axolotls, and spiny mice, provide valuable insights into the molecular basis of kidney regeneration. The identification of conserved and divergent injury responses suggests evolutionary conservation in core AKI mechanisms, while also pointing to regeneration-associated transcriptional programs that could inform future therapeutic strategies. This work underscores the importance of using non-traditional models as well as the value in comparative analysis with traditional models and clinical data.

## Introduction

Acute kidney injury (AKI) remains a major clinical challenge, characterized by a rapid decline in glomerular filtration rate, accumulation of metabolic waste, and disruption of fluid and electrolyte homeostasis. Worldwide, AKI affects up to 20% of hospitalized patients and carries a high mortality rate in its most severe forms (Susantitaphong et al., 2013; Kellum et al., 2021). Even when patients survive the initial injury, AKI is known to result in incomplete recovery with respect to renal function. Even a single episode of AKI predisposes many and accelerates the progression of chronic kidney disease (CKD), resulting in diminished quality of life for patients (Ishani et al., 2009; Zuk and Bonventure, 2019; Kurzhagen et al., 2020). This creates a pressing need for interventions that foster true renal regeneration rather than merely mitigating acute damage.

Mammalian kidneys, including those of humans and mice, often possess a limited capacity for regeneration/healing post-injury. After AKI, repair mechanisms in these systems predominantly involve proliferation of surviving tubular epithelial cells and activation of fibrotic pathways, rather than the *de novo* nephron formation, or neonephrogenesis (Yang et al., 2010; Jin et al., 2020; Yu and Bonventre, 2020). As a result, traditional mammalian models, while invaluable for understanding injury pathways and development of novel therapeutics, fall short in revealing the molecular programs that underpin full structural and functional restoration of renal tissue.

In contrast to these traditional mammalian models, the spiny mouse (*Acomys*) has the unique ability to regenerate renal tissue among others, without developing fibrotic/scarred tissue in comparison to traditional laboratory mice (Jiang et al., 2019; Okamura et al., 2021). Upon injury via unilateral ureter obstruction, the spiny mouse downregulates profibrotic cytokines in comparison to C57/BL6 counterparts (Okamura et al., 2021).

Other, non-mammalian models such as the zebrafish (*Danio rerio*) and the axolotl (A*mbystoma mexicanum*) have also provided crucial insights into regeneration. Zebrafish have greatly emerged as a powerful, high-throughput model for studying kidney regeneration as adult zebrafish are capable of robust epithelial repair within individual nephrons which occurs concomitantly with neonephrogenesis (Zhou et al., 2010; Diep et al., 2011; McCampbell et al., 2015; Brilli et al., 2019; Liu et al., 2023). These parallel processes ensure rapid recovery from catastrophic organ damage. While there have been a number of advances in understanding the underlying molecular mechanisms that accomplish neonephrogenesis, the process is still not completely understood (Chiba et al., 2016; Brilli et al., 2019; Liu et al., 2023; He et al., 2025).

Research using the axolotl, a neotenic salamander, is in its infancy (Chen et al., 2023). However, theaxolotl model has a long history of utility for studying mechanisms of vertebrate regeneration due to their ability to replace complex body parts such as their limbs, tail, spinal cord, lenes and brain, with recent studies exacting cellular niches with differential expression patterns (Wigmore and Holder, 1985; Lust et al., 2022).

As a whole, high-throughput transcriptomic profiling has greatly enhanced our understanding of the transcriptional networks driving injury response, repair, and development in the kidney (Lindström et al., 2021; Luft, 2021; Hinze et al., 2022; Creed et al., 2024). Notably, the recent meta-analysis conducted by Abdank et al. systematically compared transcriptomic signatures across multiple human and mouse AKI studies, uncovering conserved and species-specific injury markers (Abdank et al., 2024).

Despite these advances, comparative studies have yet to incorporate zebrafish, spiny mouse, and axolotl into a unified cross-species meta-analysis. To address this gap, our study builds upon the foundational work of Abdank et al. by integrating recently published RNA-seq datasets of zebrafish, spiny mouse, and axolotl kidney regeneration with two human and two murine AKI datasets (Okamura et al., 2021; Chen et al., 2023; He et al., 2025). While we were unable to match injury methodologies across all species, due to species specific limitations when using aquatic-based animal models, we hope to highlight conserved transcriptional patterns found in regenerating tissues (with some commentary at the single cell level) as well as explore the transcriptional landscape across species in AKI and its subsequent recovery process across species and notable injury models.

## Methods

### Data summary

All data analyzed in this study were obtained from previously published sources (Table 1; Supplementary Table S1).

**TABLE 1:** Summary information of each publication from which data was analyzed in this study with number of biological replicates.

			Replicates		
Publication	Species	AKI Model	AKI	Recovery	Healthy
Hinze et al., 2022	Human	Infection	8	na	4
Lake et al., 2023	Human	Varied	22	na	58
Balzer et al., 2022	Mouse	IRI	8	2	6
Kirita et al., 2020	Mouse	IRI	12	na	4
He et al., 2025	Zebrafish	Gentamicin	6	6	3
Chen et al., 2023	Axolotl	Gentamicin	2	na	3
Okamura et al., 2021	Spiny Mouse	UUO	3	3	3

As with injury models, the ability to find temporally consistent samples across studies is not feasible due to the limited data in non-traditional model species. Therefore, we refer to AKI in our analysis as the first sampling after the initial injury. We refer to recovery as either recovery defined by Abdank et al. (for the Balzer et al., 2022 data), the second (and/or last) sample taken (for the Okamura et al. and He et al. data) (Supplementary Table S1).

### Raw data processing and estimation of the count matrix

Estimation of the count matrix was derived from the FPKM matrix found within the Supplementary Material of He et al. (2025). In order to compute the estimated count matrix, we randomly sampled the mapped reads from. fastq files, computed the mean number of mapped reads (108,332,354, sd: 18,008,164), the mean alignment rate (25.43% sd: 1%), and the length of each given gene. Mapping was performed via HISAT2 (V2.1.0) utilizing the zebrafish reference genome (GRCz11). We then estimated the number of reads of a given transcript ‘ $\hat{C_{i}}$ ’ by multiplying the FPKM of a transcript ‘ ${FPKM}_{i}$ ’ by the mean number of mapped reads by the length of the gene of interest ‘ ${kb}_{i}$ ’. This product was then divided by one million (Equation 1).

$${\hat{C}}_{i}=\frac{{FPKM}_{i}\times \bar{MR}\times {kb}_{i}\;}{10^{9}}$$ (1)

### Bulk RNA seq analysis and GO-enrichment

All bulk RNA seq analysis was carried out in R (V4.4.0) utilizing the ‘DESeq2’ package (V1.46.0). For gene-ontology (GO) analysis, we utilized the ‘clusterProfiler’ package (V4.14.4). For GO-enrichment, we utilized Benjamini and Hochberg correction for pairwise comparisons and set both the p- and q-value thresholds for GO-terms to p/q < 0.05.

### Mapping zebrafish and spiny mouse genes to human orthologs

As we utilized the human orthologs for mouse transcripts provided from the meta-analysis performed by Abdank et al., we were left with the determination of the human orthologs for the zebrafish and spiny mouse transcriptome. For this mapping we utilized the ‘biomaRt’ package (V2.62.1) to access/map zebrafish genes to the human ortholog (Zebrafish: org. Dr.e.g.,.db, V3.20.0; Human: org. Hs.e.g.,.db, V3.20.0). Notably, we did refer to the mouse (*Mus musculus*) genome in our mapping of the spiny mouse transcripts to the human genome which was also performed via ‘biomaRt’ (org.Mm.eg.db, V3.21.0).

### Batch effect corrections

To account for potential batch effects derived from different studies, we utilized a mixed-effects model paradigm aimed at isolating transcript-level differential expression patterns. For each gene, a random effect was assigned at the paper level, with the condition (AKI/Health/Recovery) being a fixed effect (Equation 2). For our mixed model approach, we utilized the lme4 package (V1.1-36) (Bates et al., 2015). To gain a holistic understanding of the data our selection criteria for further analysis of mixed-effects models was p < 0 0.1.

$$\log_{2}\left(fc\right)∼\text{condition}+\left(1\;|\;paper\right)$$ (2)

To provide a formal assessment of batch correction via our mixed-effects model approach, we performed principal component analysis (PCA) on the normalized predictions from the model. Naturally, as not all genes would be expressed due to mapping, read depth, or other technical/biological variations, we only utilized transcripts that appeared in 60% of studies/conditions. For the instances where expression of a given gene was not found, we utilized the numeric mean across the available datasets for that transcript, allowing us to avoid artificial inflation of condition associated variance. Within the PCA plot, due to the innate biological based variation found across species and conditions, we only looked for separation of paper level variance.

## Results

### The homo- and heterogeneity of differentially expressed genes in response to AKI and in healthy tissue

**FIGURE 1:**
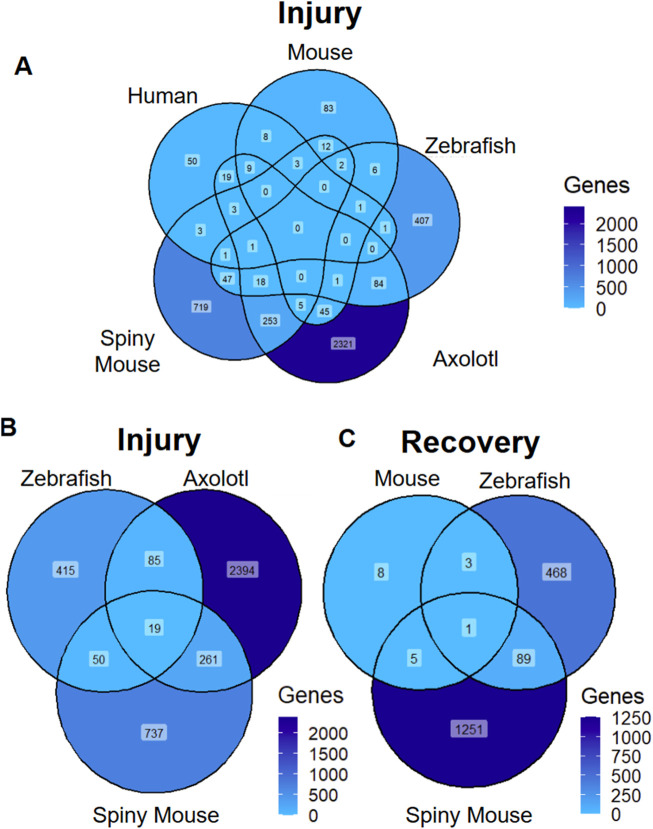
Differentially Expressed Transcripts Across Species and Injury Models **(A)** AKI differentially expressed markers across species **(B)** ‘Injury’ associated differentially expressed genes across species with regenerative potential **(C)** ‘recovery’ associated differentially expressed transcripts across species. ‘Genes’ refers to number of genes differentially expressed (|log_2_ (expression)| >1).

We first sought out to compare the transcriptomic data across the seven manuscripts via determining shared differentially expressed genes across species. We set a |log_2_ (expression)| cutoff for differentially expressed genes at 1 to ensure maximal amount of data for downstream analysis. We then compared the number of differentially expressed genes in human, mouse, axolotl, spiny mouse and zebrafish data in the AKI model, where we found a lack of homology between zebrafish, spiny mouse, axolotl and the mouse and human AKI counterparts (Figure 1A). In our cursory analysis of animals with the ability to regenerate, we found a fair amount of homology across the three species, where the axolotol and spiny mouse shared the greatest in common with respect to transcripts differentially expressed in post-injury animals (Figure 1B). In a second superficial analysis of transcriptional profiles found in recovery, we found that zebrafish and spiny mouse had a moderate degree of homology between the two species, especially in comparison to mouse (*Mus*) recovery, an incomplete form of recovery (Figure 1C) (Fu et al., 2018; Scarfe et al., 2019).

**FIGURE 2:**
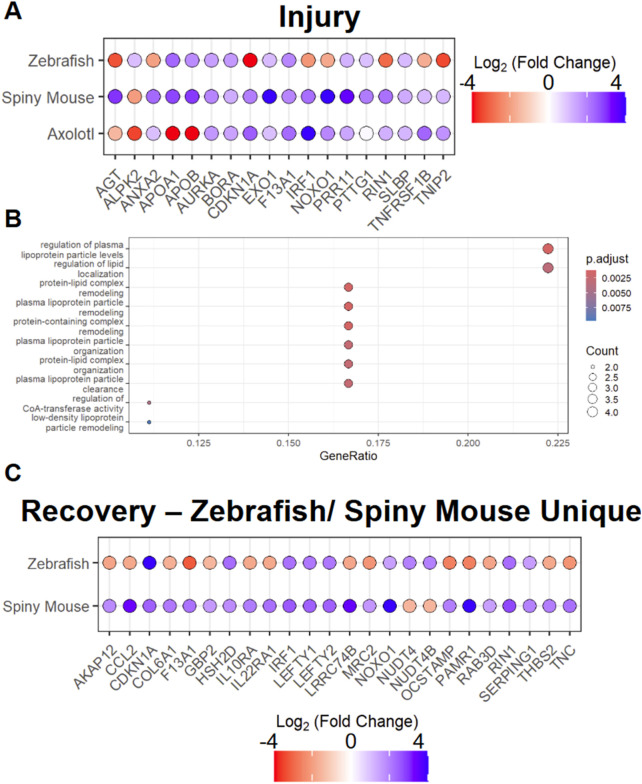
Analysis of Species with enhanced regenerative potential in injury and recovery setting **(A)** AKI associated differentially expressed markers across species **(B)** Top 10 GO terms associated with differentially expressed transcripts found in AKI response in spiny mouse, zebrafish, and axolotl, where ‘p.adjust’ is derived from Benjamini & Hochberg procedure and ‘Count’ is derived from the number of genes from Figure 2A that exist in the GO term listed on the Y-axis **(C)** ‘recovery’ associated differentially expressed genes in zebrafish and spiny mouse. *Fold changes are shown with respect to individual species expression (Ex. Spiny Mouse log_2_(FC) shown in the comparison of Spiny mouse ‘AKI’ vs. Spiny mouse ‘healthy’).

To examine the homogeneity of transcripts found in the AKI response in zebrafish, axolotl, and spiny mouse, the three species with regenerative capacities, we examined the 19 transcripts that are all differentially expressed, utilizing the same log_2_(FC) cut-ff previously utilized (|log_2_(FC)| >1) (Figure 2A). In this analysis we found that large swaths of these differentially expressed genes are conserved with respect to their differential expression pattern across species. Interestingly, we found a divergence in this pattern in the apolipoproteins among other transcripts, notably APOA1 and APOB, molecules necessary for proper development (Babin et al., 1997; Templehof et al., 2021). Other transcripts, such as CDKN1A were found to be negatively regulated in spiny mouse and axolotl, but sharply positively regulated in the zebrafish data. To investigate these 19 transcripts further, we pursued GO (gene ontology) enrichment, using a FDR of <0.05, where we found lipoproteins to be the majority of the GO terms enriched for (Figure 2B). We then also followed up on our analysis of transcripts found only in zebrafish and spiny mouse regeneration/recovery (exclusion of mouse), where we identified mixed convergent and divergent patterns in differential expression (Figure 2C). In this set, we find diverging expression patterns in genes such as CCL2 and convergent patterns in the likes of LEFTY1/2, molecules necessary for differentiation of progenitor populations (Kim et al., 2014). At the single-cell level in the human AKI model, we find that CCL2 has a cell-specific differential expression pattern, where the pro-inflammatory chemokine is upregulated within a subset of fenestrated endothelial cells (Supplementary Figure S1A–D). In subsequent GO enrichment analysis of these zebrafish and spiny mouse specific recovery transcripts, we found a strong immune response presence, especially with respect to leukocyte and granulocyte migration (Supplementary Figure S2).

**FIGURE 3:**
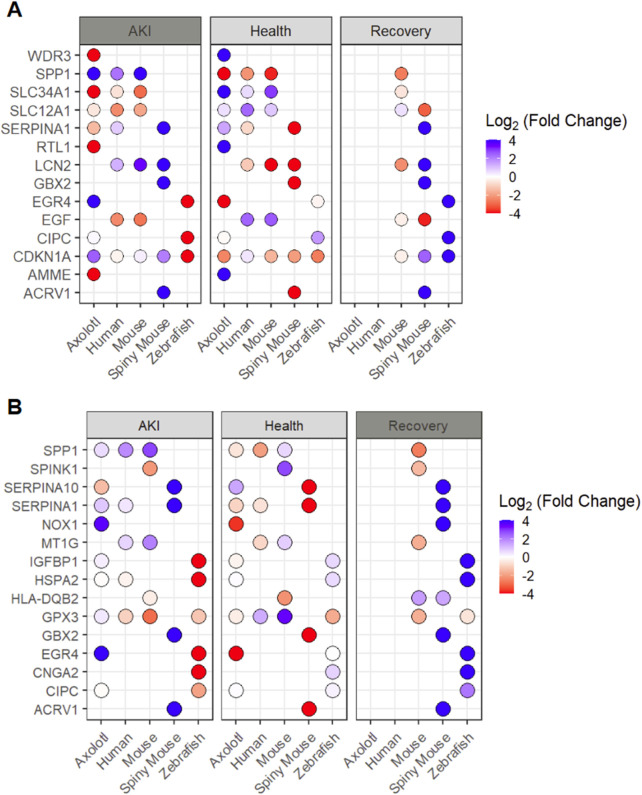
Analysis of top differentially expressed transcripts from each species (by setting) **(A)** Top 3 genes differentially expressed by each animal in AKI setting shown in all three injury setting **(B)** Top 5 genes differentially expressed by each animal in the recovery setting shown in all three injury settings and all 5 species. *Missing dot signifies transcript was not found for a given condition/species **Fold changes are shown with respect to individual species expression (Ex. Spiny Mouse log_2_(FC) shown in the comparison of Spiny mouse ‘AKI’ vs. Spiny mouse ‘Healthy’).

To examine the heterogeneity of transcriptional responses to AKI, we then set out to determine the top 3 differentially expressed genes (positively or negatively regulated) in response to AKI from each animal model. In our analysis of these potential markers for AKI, we find only one instance in which we have data for all 5 species, the cell cycle regulator CDKN1A (Figure 3A). In the expression pattern of CDKN1A, we surprisingly find downregulation of the cell cycle regulator in axolotl and spiny mouse animals, while humans and mice (*mus*) exhibited no differential expression patterns. Zebrafish however, exhibited an upregulation in *cdkn1a* in response to AKI (Figure 3A). In this analysis, we also found evidence for divergence in the transcriptional landscape in the post-AKI paradigm in zebrafish and axolotl, two species capable of regeneration. Expression of CDKN1A, CIPC, and EGR4 all showed differential expression with respect to one another, offering interesting insights in cell cycle regulation and circadian rhythm control, of which the latter has already been recently investigated (He et al., 2025; He and Sun, 2025). To further our understanding of the cell cycle regulator CDKN1A, we looked for single-cell resolution based transcriptional shifts, and found that similarly to CCL2, CDKN1A is upregulated in fenestrated endothelium in the AKI model (Supplementary Figure S3A–D). In the recovery paradigm in the spiny mouse, we saw that transcripts often held similar expression patterns to their post-AKI counterparts (Figure 3A).

In a further analysis of transcripts associated with regeneration, we explored the top 5 differentially expressed genes in the ‘recovery’ data from each of the three species with available data and explored these transcripts across all 5 species analyzed (Figure 3B). In this analysis, we found a lack of homology across recovery transcripts with the exception of GPX3 being upregulated in both mouse (*Mus*) and zebrafish, and HLA-DQB2 being downregulated in both mouse and spiny mouse (Figure 3B). To determine the granularity of GPX3 expression in the human AKI model, we looked at the single cell data provided by Hinze et al., where we find that GPX3 is upregulated in the AKI model within proximal tubule populations (Supplementary Figure S4A–D). Notably, we detected a high degree of similarity in expression patterns in spiny mouse ‘recovery genes’ in comparison to their ‘AKI’ counterparts, an observation that can be seen in the original analysis (Okamura et al., 2021).

To provide a more robust approximation of cross-species transcriptional changes in both the AKI and recovery statuses, we utilized a mixed-effects modeling approach, allowing us to attempt to control for study/paper level variation (Supplementary Figure S5). From this analysis we found in AKI (with respect to Health), transcripts such as epidermal growth factor (EGF) and DUSP2 were both found to be downregulated (Supplementary Figure S6A). With respect to recovery, we find a DUSP2 to be upregulated with respect to healthy tissue, as well as PER1, among other transcripts (Supplementary Figure S6B).

### Characterization of zebrafish unique differentially expressed genes in AKI setting

**FIGURE 4:**
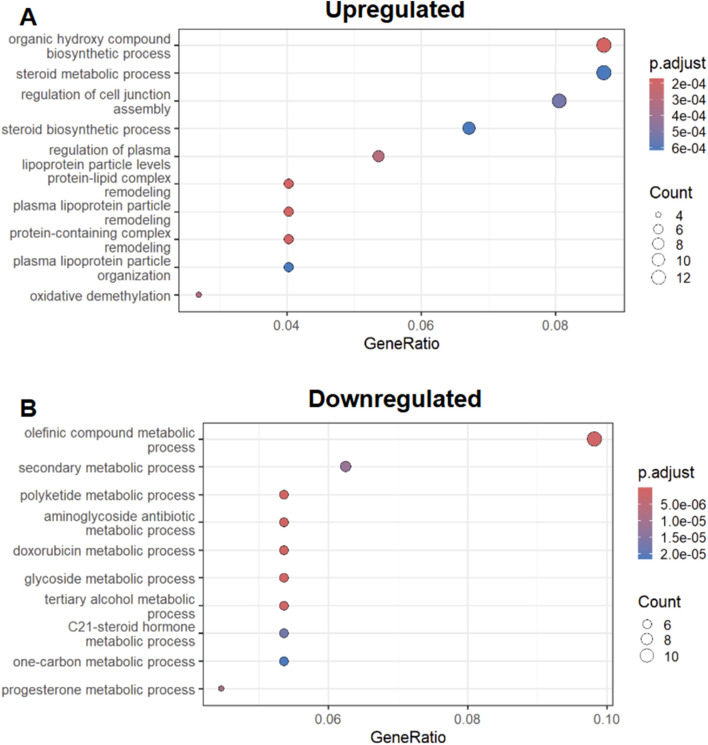
Zebrafish Specific AKI response GO enrichment **(A)** Top 10 GO terms associated with upregulated zebrafish specific AKI transcriptional response. **(B)** Top 10 GO terms associated with downregulated AKI transcriptional responses. * ‘p.adjust’ is derived from Benjamini & Hochberg procedure and ‘Count’ is derived from the number of genes from Figure 2A that exist in the GO term listed on the Y-axis.

As we found a lack of homogeneity with respect to transcriptional responses to injury and recovery, we then went to analyze transcripts only found in the zebrafish AKI model. We performed GO (Gene Ontology) enrichment on upregulated transcripts found in this zebrafish (only) AKI (only) subset. We found a diverse array of molecular processes were found to be upregulated in this subset analysis, with steroid and plasma lipoprotein regulation being among the top GO-terms (Figure 4A).

In our analysis of downregulated AKI/zebrafish only transcripts we found a similar case in the analysis of upregulated GO-terms, with diverse biological and metabolic processes being associated with the subset (Figure 4B). However within this downregulated GO-term set, we found prostaglandin metabolism terms were associated with the downregulated subset (Supplementary Table S2).

Regeneration and development are often thought to go hand-in-hand, with processes in each often mirroring each other. Therefore, we queried AKI associated transcripts in zebrafish (bulk) RNA data ranging from zygote to 5 days post fertilization (dpf). We performed this analysis by visualizing AKI-associated (upregulated) transcripts that are highly expressed through development (Supplementary Figure S3A). Interestingly, we found that apolipoproteins, ferritin associated scripts, and tubulin transcripts were among those most highly upregulated transcripts found in development (Supplementary Figure S7A). Through performing a similar analysis of downregulated transcripts during development, we found varied biologically relevant transcripts dynamically expressed during development (Supplementary Figure S7B). Among these, GPX4, which has been implicated as a promising marker in the progression of advanced forms of diabetic kidney disease (Wang et al., 2022).

## Discussion

This study expands the cross-species analysis of acute kidney injury (AKI) by incorporating transcriptomic data from three species capable of renal regeneration: zebrafish, axolotl, and spiny mouse, into a comparative framework that previously centered on human and murine models (Abdank et al., 2024). Through this expanded analysis, we reveal patterns of both conserved and divergent gene expression across species in response to kidney injury and recovery, underscoring the complexity and heterogeneity of renal repair programs across species/models used in AKI studies.

Our findings reinforce the notion that regenerative species employ transcriptional programs that are distinct from those observed in traditional mammalian models. Notably, zebrafish, axolotl, and spiny mouse share a set of 19 genes that are differentially expressed during AKI, two of which are associated with apolipoproteins, items necessary for cholesterol maintenance (APOA1 and APOB). Subsequent GO enrichment of these 19 genes suggests that modulation of lipid handling and membrane repair may play a pivotal role in enabling regeneration. This comes as apolipoprotein-L1 (APOL1) has become of great interest in the nephrology community as genetic mutations in APOL1 are associated with CKD progression in humans, especially in individuals of African descent (Genovese et al., 2010; Privratsky et al., 2020). With respect to APOA1 and CKD, patients with higher serum levels of APOA1 have been associated with lower prevalence of CKD (Goek et al., 2012). In zebrafish, *apoa1a* has been investigated in larvae as a regulator or renal function, where loss of *apoa1* was associated with limited renal clearance of a sugar-fluorophore conjugate (Kotb et al., 2016). In axolotl, apolipoproteins (notably *apoeb*), have been shown to be upregulated in (primarily) cells with hematopoietic lineages during the wound repair process in limb amputation, a similar trend to which we present, where *apoeb* is upregulated in the axolotl AKI response (Leigh et al., 2018). In contrast, both zebrafish and spiny mouse show downregulation of APOB and APOA1 in the AKI context.

In our analysis of transcripts that are uniquely shared between recovery in the spiny mouse and recovery in zebrafish, we find the cell cycle regulator CDKN1A to be downregulated in both species. In the (human) AKI setting, we also found CDKN1A to be upregulated in fenestrated endothelium, suggesting a role of the kinase in the vasculature-based injury response in mammals (Basile et al., 2001; Maringer and Sims-Lucas, 2016). In a cross-species cisplatin induced AKI model, CDKN1A downregulation was found to be associated with increased cell viability and decreased presence of ferroptosis markers (Gao et al., 2024). In a mouse IRI (ischemia reperfusion injury) based transcriptomic analysis, Cdkn1a expression was found to be upregulated post injury (He et al., 2022). This suggests a potential for innate responses to injury in these species allowing for suppression of canonical injury responses to aid in the process of regeneration, albeit in a species-specific manner. Notably, in the adult axolotl pancreas, Cdkn1a is naturally downregulated in comparison to human CDKN1A, but shares a similar differential expression pattern to the mouse (*Mus*) Cdkn1a (Ma et al., 2025). Also interestingly, we found that LEFTY1/2 were both downregulated in spiny mouse and zebrafish in the ‘recovery’ stage. Supplementation of Lefty-1 in mice has been associated with a decrease in sustained injury post AKI, and has been postulated to be modulator of the immune response after injury (Xu et al., 2016; Zhang et al., 2018). With respect to GO terms associated with genes associated with both spiny mouse/zebrafish AKI, we find that, perhaps unsurprisingly, immune responses to injury, are common, with varied leukocyte properties being highlighted. Of note in immune related responses to injury, CCL2 was found to be downregulated in the spiny mouse while upregulated in zebrafish. In a skin-based spiny mouse study, *Acomys* Ccl2 was found to have no differential expression in comparison to other *Mus* species, which had upregulated Ccl2 in response to injury of the outer-ear skin (Gawriluk et al., 2020). In our analysis of human single-cell resolution data, we found that CCL2 was upregulated in endothelial populations post-injury, an idea consistent with Kirita et al., where they demonstrated endothelial cells play a role in early injury repair via CCL2 secretion (Kirita et al., 2020).

In our investigation of genes differentially expressed across all five species analyzed in this study, we found a perhaps unsurprising lack of homology across all species, with mouse and human being the most homologous in both the AKI and Health settings. From our analysis of these top differentially expressed transcripts we find evidence of homology and heterogeneity between species with regenerative capacity and those without. In the analysis of SERPINA1, a serine protease inhibitor, we find that axolotl and humans have differing expression patterns, while the spiny mouse has a similar expression pattern to that of humans (downregulation). Differing SERPINA1 expression levels has promise as a marker in those undergoing treatment for diabetic kidney disease (Ahluwalia et al., 2024). Of the isoforms of SERPINA1, transcripts derived from latter portion of the first exon are disproportionately expressed in the kidney, with respect to upstream regions of the coding region (Matamala et al., 2015). Another example of homology across species of differing regenerative capacity can be found in LCN2, with human, mouse, and spiny mouse all showing downregulation of the transcript. In the mouse, inhibition of LCN2 can lead to exacerbation of phenotypes in unilateral ureteral obstruction models (Qiu et al., 2018). Interestingly, these same results from Qiu et al. show that Lcn2 accumulates after UUO injury, a finding at odds with the IRI injuries in which were used to generate the data analyzed within this study (Qiu et al., 2018).

In our complimentary analysis, where we surveyed highly differentially expressed transcripts in recovery across ‘injury’ and ‘health’, we find relative homology in genes such as GPX3, where GPX3 is upregulated in recovery and in the post-injury paradigm in the human, zebrafish, and mouse (*Mus*) models, while (slightly) downregulated in axolotl. When we refined our lens to look at cellular level expression pattern changes in the AKI model, GPX3 was found to be upregulated in proximal tubule populations, perhaps unsurprisingly, as the proximal tubule is the primary source of GPX for the body, and has been implicated as having a protective role in other ailments (Avissar et al., 1994; Tham et al., 2002; Olson et al., 2010) In rat, Gpx3 has been validated as a marker for ischemia-reperfusion injuries, the injury model used in the mouse studies analyzed in this analysis (Pei et al., 2023). In an earlier IRI study, also using rat, Gpx3 was downregulated, demonstrating further transcription level divergence across animal models (Wu et al., 2023). We also once again find a re-emergence in our analysis of SERPINA1 and SERPINA10, where the markers are downregulated in both the AKI and recovery paradigms in the spiny mouse, while being upregulated in the AKI paradigm in axolotl. Finally we look to MT1G, which was unregulated during incomplete recovery in mice (*Mus*) and in healthy states in both mammals but was not differentially expressed in zebrafish, suggesting a perhaps nuanced, but not mammalian specific role in renal repair or homeostasis. However, in ccRCC, MT1G has been implicated in having a potential protective role from ferroptosis via negative regulation of glutathione metabolism (Zhang et al., 2022).

In our mixed models approach at surveying global trends in transcriptional alterations in the AKI model, we found that epidermal growth factor (EGF) to be significantly downregulated. EGF has previously been indicated as a regulator of recovery in the post-AKI setting, but has also been notably characterized as a marker for CKD progression (Chen et al., 2012; Ju et al., 2015; Klein et al., 2016). With respect to recovery-associated transcripts, we found that DUSP2 was upregulated, an interesting contrast to its upregulation in the ‘injury’ model. In mice, loss of DUSP2 was associated with exacerbation of AKI phenotypes, while gain of expression lessened injury (Xiong et al., 2022). Interestingly within this recovery set, we find significant upregulation of PER1. PER1, one of the key regulators of the circadian rhythm, has only recently been appreciated, along with other circadian rhythm associated transcripts, as having a dynamic and vital role in renal health (Douma et al., 2022; Zietara et al., 2022; He et al., 2025)

To better characterize zebrafish-specific transcriptional responses, we performed GO enrichment analysis on transcripts uniquely upregulated or downregulated during zebrafish AKI. These analyses revealed involvement of pathways related to lipid metabolism, steroid processing, and prostaglandin signaling. Notably, prostaglandin signaling has been of great interest in both renal regeneration and development in zebrafish, as well as in humans (Nørregaard et al., 2015; Poureetezadi et al., 2016; Liu et al., 2023). In a follow up of these zebrafish-specific transcriptional regulators, we also find that these genes found in the AKI response can also be found to have dynamic expression patterns in development. This comes at no surprise, as previous studies have shown that transcriptional regulators of renal development can play active roles in AKI (Wingert et al., 2007; Chiba et al., 2015; Yang et al., 2023).

In conclusion, our findings emphasize the value of incorporating non-traditional model organisms into AKI studies. These regenerative species not only reveal conserved pathways that may be critical for repair but also uncover alternative strategies that can inform new approaches. Future studies integrating spatial transcriptomics and proteomics across all species will only further expand our understanding of the cellular and molecular basis of kidney regeneration.

## Data Availability

Publicly available datasets were analyzed in this study. This data can be found here: See Table 1 in Manuscript for appropriate citations.

## Abbreviations

AKI, acute kidney injury; ccRCC, clear cell renal cell carcinoma; CKD, chronic kidney disease; EGF, epidermal growth factor; GO, gene ontology; IRI, ischemia reperfusion injury; scRNA-seq, single cell RNA sequencing; UUO, unilateral ureteral obstruction.
