# Supplementary material for: A comparative transcriptomics analysis of mammalian and non-mammalian acute kidney injury (AKI) models
Source: Front Cell Dev Biol. 2025 Sep 26;13:1653967. doi: 10.3389/fcell.2025.1653967 (PMC12510925; doi:10.3389/fcell.2025.1653967)
Supplement: Supplementary file 1 [file Supplementaryfile1.docx]

**Supplemental Information**

**
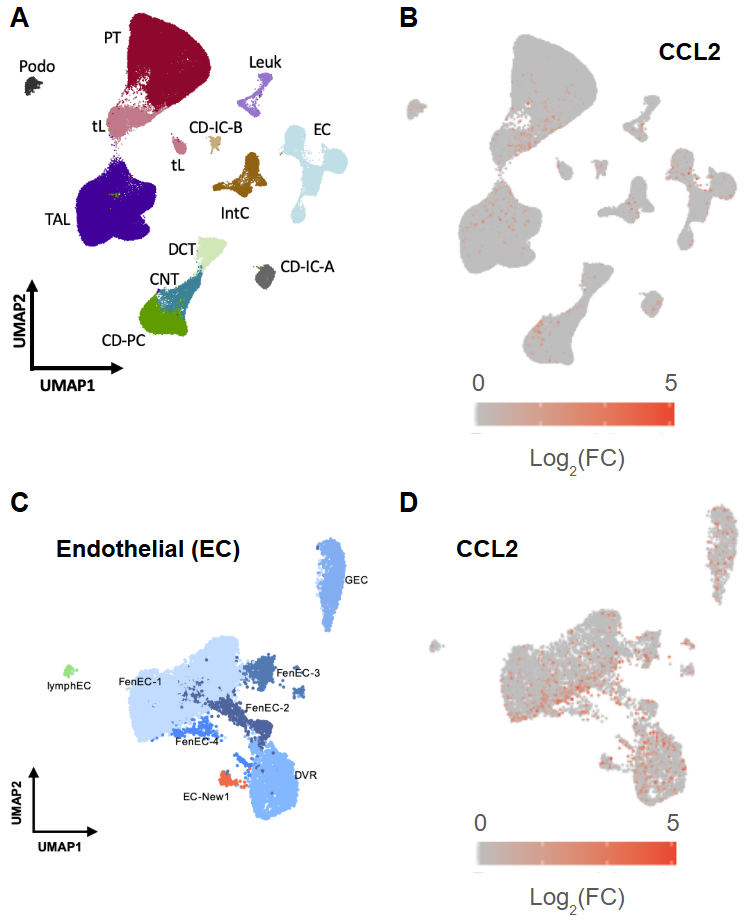
**

Supplemental Figure 1. Single Cell Resolution of CCL2 in human AKI model. **A)** UMAP with distinct cell populations color coded **B)** CCL2 expression across individual cell populations **C)** UMAP with distinct endothelial cell sub-clusters color coded **D)** CCL2 expression pattern in endothelial populations.

**
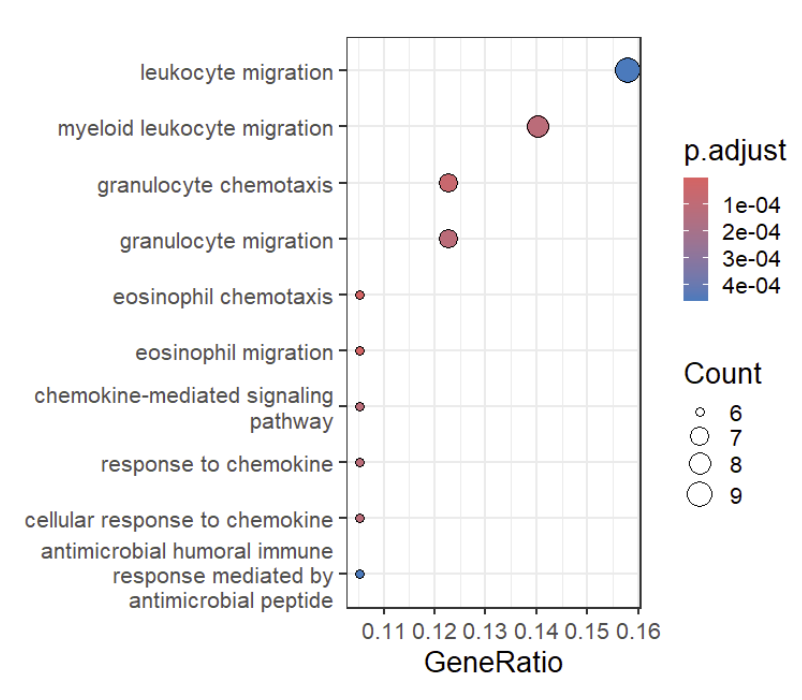
**

**Supplemental Figure 2. Zebrafish and spiny mouse specific transcripts in recovery setting associated GO enrichment** *‘p.adjust’ is derived from Benjamini & Hochberg procedure and ‘Count’ is derived from the number of genes from Figure 2A that exist in the GO term listed on the Y axis

**
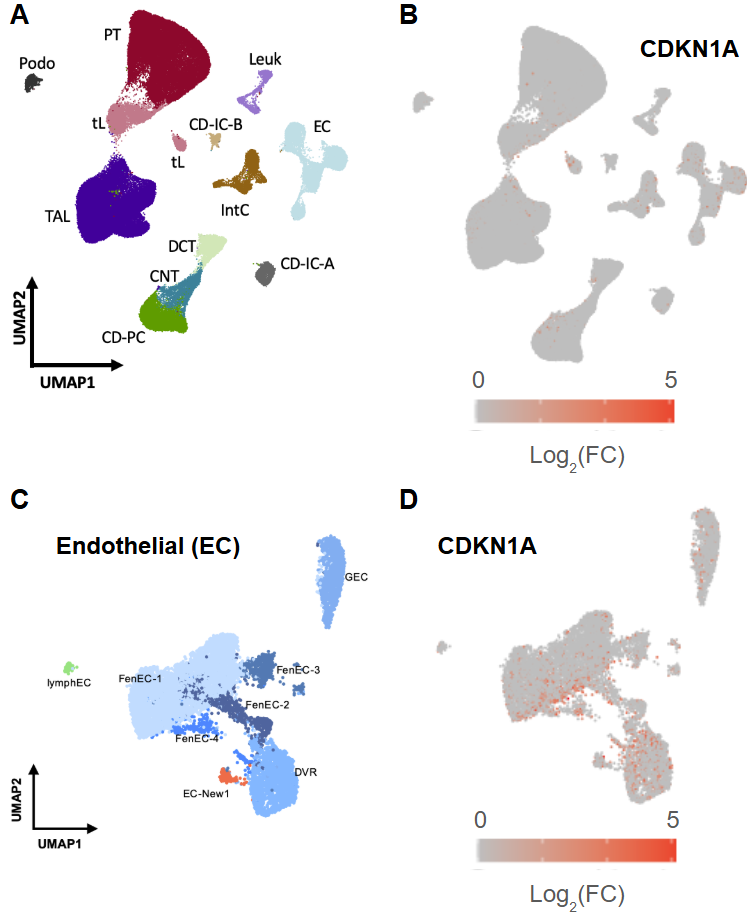
**

**Supplemental Figure 3. Single Cell Resolution of CDKN1A in human AKI model**. **A)** UMAP with distinct cell populations color coded **B)** CDKN1A expression across individual cell populations **C)** UMAP with distinct endothelial cell sub-clusters color coded **D)** CDKN1A expression pattern in endothelial populations in AKI model.

**
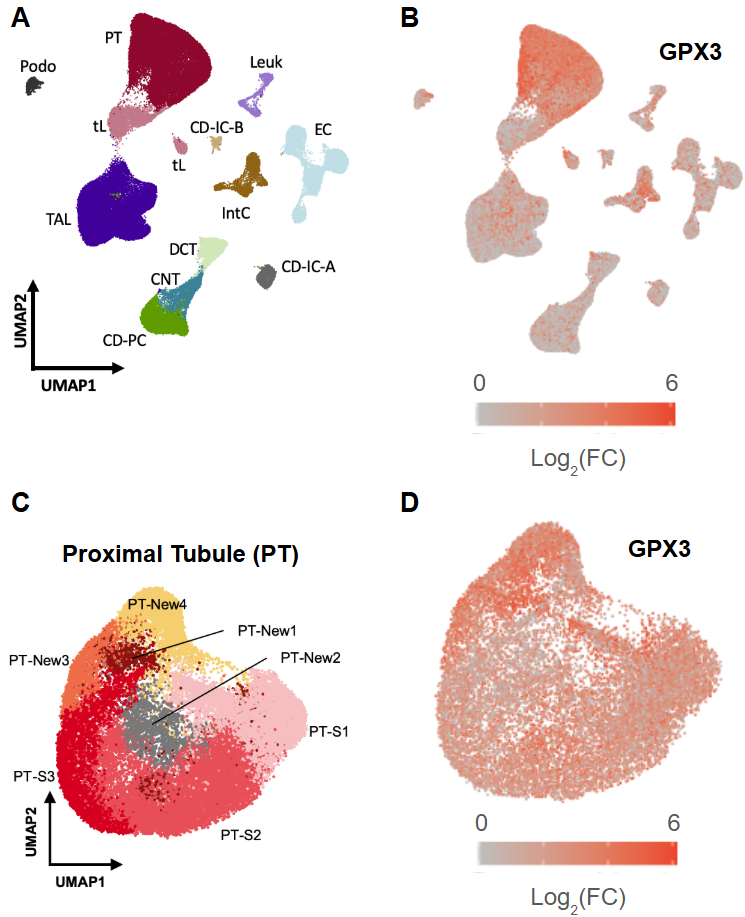
**

**Supplemental Figure 4. Single Cell Resolution of GPX3 in human AKI model**. **A)** UMAP with distinct cell populations color coded **B)** GPX3 expression across individual cell populations **C)** UMAP with distinct proximal tubule sub-clusters color coded **D)** GPX3 expression pattern in proximal tubule subclusters in the AKI model.

**
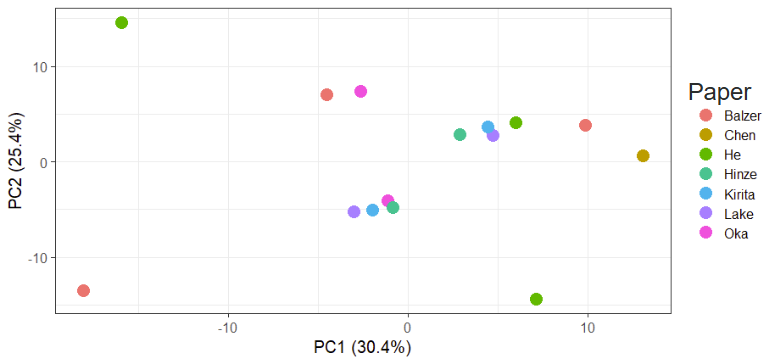
**

**Supplemental Figure 5. Principal Component Analysis of Studies of Data Origin to Determine Efficacy of Batch Effect Correction via Mixed Effect Model Approach.**

**
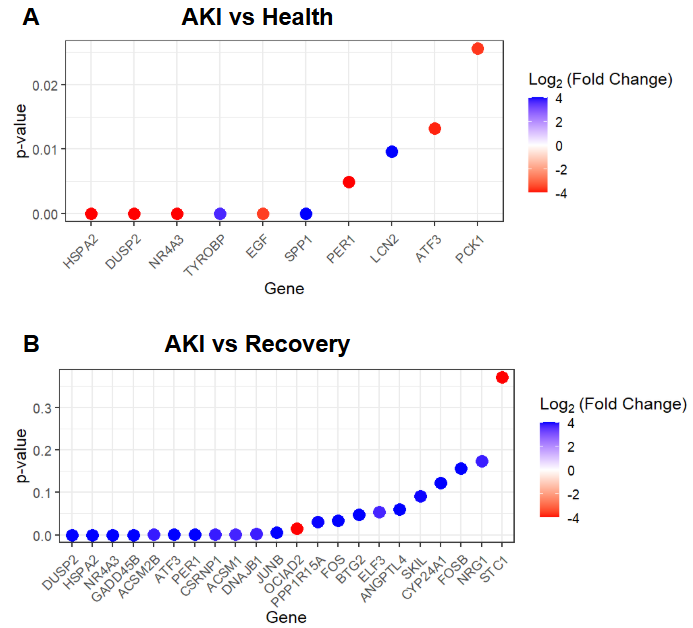
**

**Supplemental Figure 6. Outcomes of Mixed-effects model analysis A)** Transcripts differentially expressed across species in the AKI setting, with respect to healthy tissue. **B)** Transcripts differentially expressed across species in the AKI setting, with respect to Recovery tissue.

**
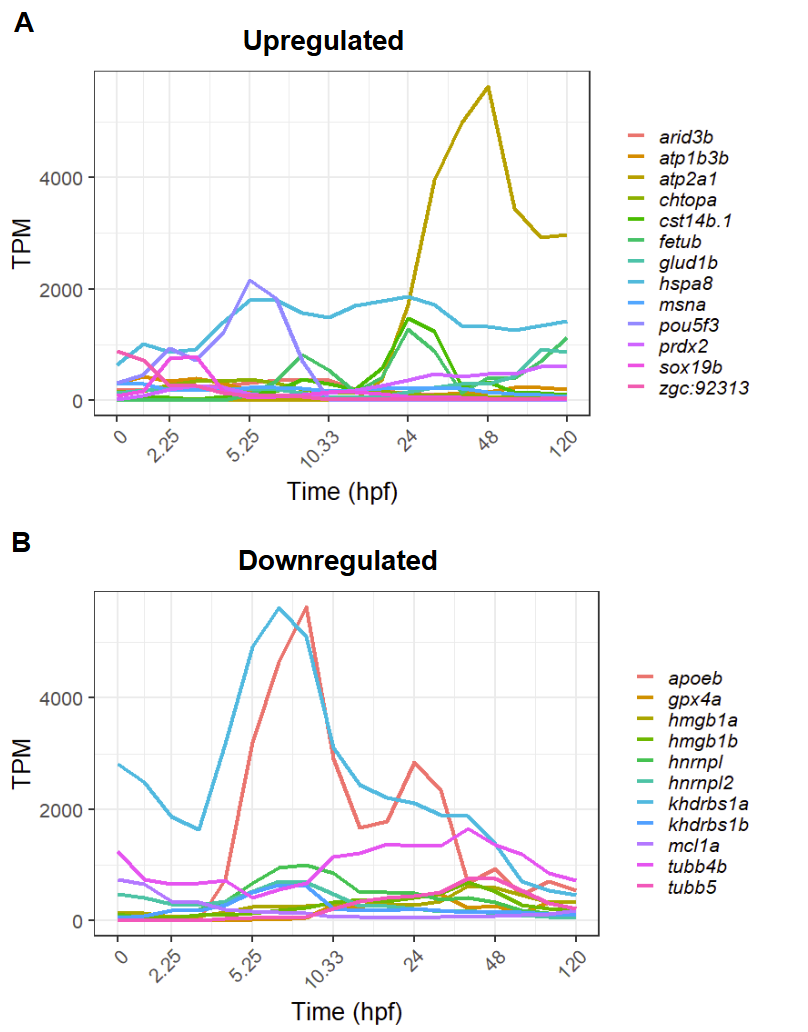
**

**Supplemental Figure 7. Zebrafish Specific AKI response transcripts in zebrafish development A)** Transcripts differentially expressed in development that are also upregulated during response to AKI in zebrafish **B)** Transcripts differentially expressed in development that are also downregulated during response to AKI in zebrafish

**
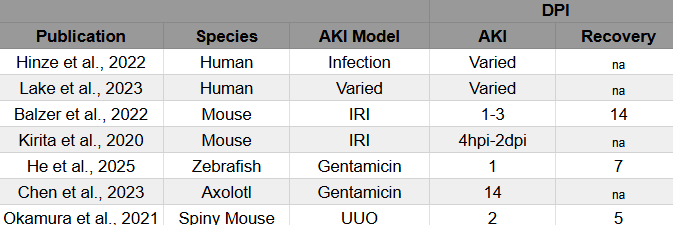
**

**Supplementary Table 1. Further classification of models and time points (post injury) used in the studies analyzed**

| aminoglycoside antibiotic metabolic process | oogenesis | regulation of keratinocyte proliferation |
| --- | --- | --- |
| C21-steroid hormone metabolic process | piRNA processing | response to toxic substance |
| doxorubicin metabolic process | polyketide metabolic process | secondary metabolic process |
| female gamete generation | primary alcohol metabolic process | steroid metabolic process |
| glycoside metabolic process | progesterone metabolic process | tertiary alcohol metabolic process |
| hormone metabolic process | prostaglandin metabolic process | regulation of keratinocyte proliferation |
| lactone metabolic process | prostanoid metabolic process |  |

**Supplementary Table 2. GO terms associated with upregulated, zebrafish specific, AKI response**
